# Hemostasis in neonatal ECMO

**DOI:** 10.3389/fped.2022.988681

**Published:** 2022-08-26

**Authors:** Valeria Cortesi, Genny Raffaeli, Giacomo S. Amelio, Ilaria Amodeo, Silvia Gulden, Francesca Manzoni, Gaia Cervellini, Andrea Tomaselli, Marta Colombo, Gabriella Araimo, Andrea Artoni, Stefano Ghirardello, Fabio Mosca, Giacomo Cavallaro

**Affiliations:** ^1^Neonatal Intensive Care Unit, Fondazione IRCCS Ca' Granda Ospedale Maggiore Policlinico, Milan, Italy; ^2^Department of Clinical Sciences and Community Health, Università degli Studi di Milano, Milan, Italy; ^3^Angelo Bianchi Bonomi Hemophilia and Thrombosis Center, Fondazione IRCCS Ca' Granda Ospedale Maggiore Policlinico, Milan, Italy; ^4^Neonatal Intensive Care Unit, Fondazione IRCCS Policlinico San Matteo, Pavia, Italy

**Keywords:** neonatal ECMO, developmental hemostasis, thrombosis, anticoagulation management, point-of-care tests

## Abstract

Extracorporeal membrane oxygenation (ECMO) is a life-saving support for cardio-respiratory function. Over the last 50 years, the extracorporeal field has faced huge technological progress. However, despite the improvements in technique and materials, coagulation problems are still the main contributor to morbidity and mortality of ECMO patients. Indeed, the incidence and survival rates of the main hemorrhagic and thrombotic complications in neonatal respiratory ECMO are relevant. The main culprit is related to the intrinsic nature of ECMO: the contact phase activation. The exposure of the human blood to the non-endothelial surface triggers a systemic inflammatory response syndrome, which chronically activates the thrombin generation and ultimately leads to coagulative derangements. Pre-existing illness-related hemostatic dysfunction and the peculiarity of the neonatal clotting balance further complicate the picture. Systemic anticoagulation is the management's mainstay, aiming to prevent thrombosis within the circuit and bleeding complications in the patient. Although other agents (i.e., direct thrombin inhibitors) have been recently introduced, unfractionated heparin (UFH) is the standard of care worldwide. Currently, there are multiple tests exploring ECMO-induced coagulopathy. A combination of the parameters mentioned above and the evaluation of the patient's underlying clinical context should be used to provide a goal-directed antithrombotic strategy. However, the ideal algorithm for monitoring anticoagulation is currently unknown, resulting in a large inter-institutional diagnostic variability. In this review, we face the features of the available monitoring tests and approaches, mainly focusing on the role of point-of-care (POC) viscoelastic assays in neonatal ECMO. Current gaps in knowledge and areas that warrant further study will also be addressed.

## Introduction

The role of extracorporeal membrane oxygenation (ECMO) in the neonatal population has been clearly established in the last decades (1). Furthermore, it has been demonstrated ECMO supports cardiac and pulmonary reversible diseases once conventional management has failed (2). In comparison, extracorporeal cardiopulmonary resuscitation (ECPR) represents a minor neonatal ECMO indication (2). In 2021 in Europe, 261 neonatal ECMO have been performed, 148 for pulmonary, 88 for cardiac, and 25 for ECPR indications (3).

Despite technological improvements, such as miniaturization and simplification of circuits, new coating systems, new vascular accesses, low hemolytic centrifugal pumps, and increasingly efficient artificial membranes, hemostatic management remains a real challenge during ECMO (4). Thrombosis and bleeding frequently occur, even in the same patient. Coagulation problems are the main leading cause of mortality and morbidity, as reported by the Extracorporeal Life Support Organization (ELSO) registry in 2021 (5). In neonatal pulmonary ECMO, the most common hemostatic complications reported are brain hemorrhage (intraventricular or intra-extra parenchymal, respectively, 2.9 and 3.3%), peripheral cannula site bleeding (4.3%), surgical site bleeding (6.3%), clots in the circuit components (29.2%), moderate or severe hemolysis (10%), pulmonary hemorrhage (3.4%), gastrointestinal bleeding (1.4%) (5).

The ideal ECMO running should consist of an “unclotted” circuit in a “clotting” patient. However, patient and circuit factors make obtaining this hemostatic balance difficult. Hence, the real ECMO running is an “unclotted” patient and a “clotted” circuit.

The exposure of human blood to the non-endothelial surface of the circuit determines platelet aggregation, continuous activation of the coagulation cascade, and the chronic generation of thrombin. Over time, this leads to coagulation problems, especially in a prothrombotic trend (6). Moreover, the continuous platelet activation results in down-regulation of the receptors and degranulation, making platelets hyporeactive and dysfunctional, representing an important cause of bleeding (6).

The neonatal hemostatic system is intrinsically unstable, being in developmental evolution in the first days and weeks of life (7, 8). This means it could be more susceptible to hemostatic alterations and less capable of facing bleeding or thrombotic triggers. Moreover, the pre-existing conditions and comorbidities that led the patient to ECMO may have triggered a systemic inflammatory response, contributing to the coagulation derangements (9–12).

Unfractionated heparin (UFH) is the mainstay of hemostatic treatment (13, 14). Hemostatic function monitoring allows for dynamically adjusting the heparin dose to maintain a correct balance between anticoagulation and bleeding (13). Regrettably, there is not a single reliable test, but a combination of different laboratory examinations helps to adjust the antithrombotic therapy (4, 14, 15). Each method measures different functional and quantitative hemostatic variables and may not be comparable straightaway: activated partial thromboplastin time (APTT), anti-factor Xa, and antithrombin (AT) levels are plasma-based tests, activated clotting time (ACT), and viscoelastic assays (thrombo-elastography/thrombo-elastometry) are performed on whole blood. Mainly, thromboembolic events frequently occur, although traditional coagulation tests are in the targeted ranges (15). In addition, reference ranges for neonatal age are lacking for some tests. All these factors contribute to the sizeable inter-institutional variability in diagnostic test use and management.

This review discusses the relative pros and cons of the different monitoring approaches. Next, we address the challenges in hemostatic diagnostics and anticoagulation management, focusing on the role of point-of-care (POC) viscoelastic assays in neonatal ECMO. We conclude by discussing current gaps in knowledge, emerging technologies, and areas that warrant further study.

## Why do we need to worry about hemostasis in neonates on ECMO?

### Patient-related factors

#### Developmental hemostasis

The concept of “*developmental hemostasis”* refers to the maturation of the coagulation system from fetal to adult life (7, 8, 16–18). Newborns have reduced levels (around 50% of adults) of most procoagulant factors, especially the vitamin-K dependent (FII, FIX, FX, FVII), factor XI, XII, prekallikrein, and high molecular weight kininogen (19–21). Moreover, anticoagulant factors vitamin K dependent (protein C and protein S) and antithrombin are also reduced. Similarly, the clot lysis is reduced (6, 12). With the reduction in pro- and anticoagulant factors, the hemostatic system of a healthy term neonate is then considered balanced (18, 22–24). On the other hand, factors VIII, XIII, fibrinogen, and von Willebrand factor (vWf) levels are similar or even increased at birth compared to adult life (21).

Primary hemostasis is also different in the neonatal age. The function is considered somewhat impaired even in a normal or slightly reduced platelet count at birth (25–27). Despite this described “hyporeactivity,” the functional tests, such as bleeding time and closure time (CT) of the platelet function analyzer (PFA-100), are often shortened in newborns (25, 28). Term newborns have CT shorter than preterm ones accordingly to the hyporeactivity of the preterm platelets (29). Primary hemostasis is maintained through higher hematocrit levels, mean corpuscular volume (MCV) of neonatal erythrocytes, vWf, and a large amount of high molecular weight multimers of vWf, which counterbalance platelets hyporeactivity (26, 27, 30).

Then, this developing hemostatic system is intrinsically susceptible to bleeding or thrombosis in critical neonates, depending on the underlying disease and the systemic response to different triggers (12). In this scenario, ECMO may further complicate the picture by disrupting this delicate balance due to the increased risk of dilutional coagulopathy, reducing thrombin generation, and rising bleeding risk (Table 1). In addition, neonates are more prone to heparin resistance secondary to low antithrombin concentrations (6).

**Table 1:** Factors contributing to hemostatic derangements in ECMO.

	**Patient-related factors**	**Circuit-related factors**
Pro-thrombotic factors	- Reduced levels of AT, protein C, protein S - high hematocrit, high MCV, high vWf, high molecular weight multimers of vWf - Pre-existing conditions (cytokines → activation of primary and secondary hemostasis, reduced action of natural anticoagulants and antifibrinolytic system) - Intrinsic resistance to heparin	- Foreign artificial surfaces → platelets adhesion and activation of the coagulation cascade - complement activation via kallikrein → inflammation - Turbulence and low flow zones → hemolysis
Pro-hemorragic factors	- Reduced levels of vitamin K-dependent coagulation factors, factors XI, XII, prekallikrein, and high molecular weight kininogen - Platelets hyporeactivity - Pre-existing conditions (thrombocytopenia in sepsis, reduced platelets production, and reduced levels of large multimers of vWf in cyanotic CHD)	- Hemodilution - Overactivation of fibrinolysis → clotting factors consumption, impaired platelet function, and thrombocytopenia - Shear stress → disruption of high molecular weight vWf multimers → “acquired von Willebrand syndrome.”

AT, antithrombin; CHD, congenital heart disease; MCV, mean corpuscular volume; vWf, von Willebrand factor.

#### Pre-existing conditions and comorbidities

A strict link between inflammatory response caused by infection or injury and activation of the coagulation cascade is well-described (9). Therefore, a disruption in one of these mechanisms may affect the entire balance, leading to excessive inflammatory response or coagulopathy (Table 1) (10).

Inflammation determines the activation of primary and secondary hemostasis in the endothelial lumen, decreases the action of natural anticoagulants, and inhibits the antifibrinolytic system through cytokine release. Also, natural anticoagulants reduce the endothelial response to inflammatory mediators (9, 11). Therefore, critically ill newborns who have systemic inflammatory response syndrome often have associated coagulopathy (12).

The baseline coagulation status and the platelet count may vary depending on the underlying pathology, as newborns affected by meconium aspiration syndrome have different hemostatic profile compared with septic or cardiopathic ones.

For example, children with cyanotic congenital heart disease (CHD) have thrombocytopenia secondary to increased destruction of peripheral platelets and reduced platelet production despite the increased erythropoietic stimulus (11, 31). The pathogenetic mechanism is not entirely clear. However, it appears to be due to the inhibition of megakaryocyte differentiation consequent to chronic hypoxia and right-to-left shunt in the Botallo arterial duct, allowing blood to bypass the pulmonary bed, where megakaryocytes break down into platelets (32–34). Furthermore, the increased risk of bleeding in these patients is due not only to thrombocytopenia but also to reduced levels of large multimers of vWf (11, 32). In addition, bone marrow suppression in sepsis or immune reactions may also determine thrombocytopenia (35).

### Circuit-related factors

#### The ECMO-circuit

All components of the ECMO circuit, such as cannulas, tubes, blood pump, and membrane oxygenator, are artificial non-biological surfaces (12, 36). Therefore, blood exposure to these foreign biomaterials activates the coagulation cascade and the inflammatory response (Table 1) (35, 37).

Some advances in technology have been obtained in the last years: 1. biomimetic tubes, with heparin-coated or nitric oxide-bonded surfaces, are thought to reduce cellular activation, release of proinflammatory mediators, and platelets activation; 2. biopassive materials for circuit lines, such as phosphorylcholine or poly-2-methoxy-ethyl acrylate (PMEA), prevent the thrombogenic response, with a reduction in platelets and complement activation; 3. endothelialization of the circuit, *in vivo* or *in vitro*, could be the future for ECMO circuits, as endothelium is the key element which physiologically regulates inflammation and coagulation within the body (4). However, these technologies still do not avoid the need for systemic anticoagulation, and they are still not widely available soon as part of ECMO circuits (4).

After the start of ECMO, plasma proteins, including albumin, factor XII, fibrinogen, and kallikrein, are immediately attached to the non-endothelial surface, followed by vWf and glycoproteins (6, 13, 36). Factor XII is then activated to factor XIIa, which activates the intrinsic pathway of the coagulation cascade, with consequent thrombin generation (6).

Von Willebrand factor binds and activates circulating platelets, producing exposure of tissue factor (TF) and activation of the extrinsic coagulation pathway (13). Kallikrein activates systemic inflammatory response via the complement system. The complement cascade determines further activation of platelets, polymorphonuclear cells, and expression of cytokines which further increase the proinflammatory state (12, 36). Fibrinolysis is then activated to limit clot formation and thrombosis (13). In reaction to the massive procoagulant state, overactivation of fibrinolysis determines the consumption of clotting factors, impaired platelet function, and thrombocytopenia (21). In neonates, contact and complement activation seem to predominate in the first 24 h, while after 72 h of ECMO start, clotting, and fibrinolytic system play the leading role (Table 1) (12, 35).

#### Inflammation and endothelial dysfunction

Proinflammatory cytokines are secreted in response to the patient's illness and exposure to the ECMO circuit. Inflammation and hypercoagulability are strictly connected: increased levels of interleukin (IL) 6 are associated with ECMO-related multiorgan failure, while some soluble cytokines (IL-6, IL-8, tumor necrosis factor-α) may contribute to the endothelial damage (36). Turbulent blood flow and increased shear stress generated by the ECMO circuit promote further cellular damage, platelet activation, and hemolysis (12, 35). Endothelial dysfunction may lead to continuous platelet and coagulation activation, resulting in consumption coagulopathy (Table 1) (12).

#### Thrombin generation

Thrombin generation results from the coagulation cascade via TF and contact system. Thrombin is central in the coagulation pathway, as it converts fibrinogen to fibrin and activates factors XIII, V, VIII, XI, and platelets. The production of a small amount of thrombin determines an explosive amplification of the entire process (12, 36). Unfractionated heparin inhibits clot formation but is not totally efficacious in inhibiting thrombin generation and coagulation within the circuit (12, 36). Moreover, neonates on ECMO showed persistent coagulation activation, represented by increased thrombin generation and fibrinolysis (Table 1) (38).

#### Platelets and von Willebrand factor

Platelet alterations involve both platelet count and platelet function. They are well-described during ECMO and may lead to bleeding and thrombotic complications (35). Thrombocytopenia may result from underlying conditions, such as sepsis, which is especially common in neonatal intensive care units (NICUs), where its incidence is 18–35% (39). In addition, hemodilution from the ECMO priming volume may exacerbate the pre-existing reduced platelet count (35). Usually, platelet count decreases >40% within the first 1–2 h of ECMO start (14).

Functional impairment is determined by platelet activation, which occurs at the start of ECMO, resulting from contact with the circuit's surface, shear stress, and turbulent flow within the tubes. Activated platelets adhere to the ECMO oxygenator and endothelium, increasing the thrombotic risk (12, 35). In addition, platelet microparticles, fragments of activated platelet membrane released during shear stress, contribute to thrombus formation as they are involved in vascular injury and promote prothrombotic and proinflammatory conditions (12, 35, 40, 41). Their procoagulant properties are mediated by membrane phosphatidylserine exposure, which activates the contact-dependent coagulation pathway. Elevated levels of microparticles are found in many thrombotic states, such as arterial thrombosis, idiopathic thrombocytopenic purpura, and thrombotic thrombocytopenia. Furthermore, platelet microparticles are involved in cell-to-cell communication, modulating innate and adaptative immunity, and cancer-related angiogenesis (40, 41).

The dysfunctional platelet activation determines platelet exhaustion and decreased aggregation, resulting from degranulation, receptor inhibition, and down-regulation. Therefore, underlying patient conditions, consumption, and hemodilution result in thrombocytopenia (36, 42). The degree of thrombocytopenia is correlated with the patient's illness severity and platelet count at the time of starting ECMO rather than with the duration (4). Often, platelet transfusions are needed to correct the severe thrombocytopenia and reduce the bleeding risk (14).

Moreover, drugs with platelet inhibitory properties, such as milrinone, nitric oxide, and histamine-2-receptor blockers, are usually prescribed during ECMO, thus potentially interfering with the hemostatic balance (Table 1) (36, 43, 44).

Shear stress determines disruption of high molecular weight vWf multimers, causing the “acquired von Willebrand syndrome” (AVWS), which consists of a functional defect in vWf, associated with bleeding (12, 35, 45, 46).

This condition is well described in patients with mechanical valves and ventricular assist devices (VADs) (47). However, literature on pediatric patients is scarce, despite evidence suggesting this phenomenon is common during ECMO (47, 48). Acquired von Willebrand syndrome might occur in 100% of the patients under ECMO support, and it is reversible at the weaning (47). Due to the pivotal role of high-weight vWf multimers in neonatal primary hemostasis, AVWS might be more pronounced in newborns than adults as they counterbalance platelet hyporeactivity (46). Therefore, diagnosis of AVWS in case of bleeding should always be considered.

#### Hemolysis

Hemolysis is increased in neonates during ECMO compared to older children and adults due to turbulent flow through the smaller cannulas; fetal hemoglobin, which makes red blood cells more susceptible to mechanical stress; elevated hematocrit, and hemoglobin concentration (Table 1) (12, 49, 50). In addition, red blood cells are destroyed by mechanical damage in the circuit pump and complement activation (14). Plasma-free hemoglobin levels >50 mg/dl are suggestive of hemolysis, even if the measurement of plasma-free hemoglobin is not standardized across centers (12, 49, 50). Hemolysis contributes to renal failure, lead to the need for circuit component change, and has been associated with thrombotic events, transfusions, and mortality (49, 51). The explanation for hemolysis complications could be the overwhelming haptoglobin and hemopexin, which bind free hemoglobin. Then, the free hemoglobin binds the endogenous nitric oxide, determining vasoconstriction (4).

### How can we achieve the hemostatic balance in neonates on ECMO?

#### Unfractionated heparin and antithrombin

During ECMO, anticoagulation is mandatory to maintain the circuit components' patency and avoid thrombosis while increasing the patient's risk of bleeding (6, 14). Therefore, continuous infusion of UFH is the standard of care in pediatric and neonatal ECMO. However, the evidence is mainly derived from adult population studies, and data on pharmacokinetics and pharmacodynamics of UFH in children and neonates are scanty (13, 14). In a survey conducted in the United States (US), 94% of the centers used continuous UFH infusion, while the remaining 6% used bivalirudin, a direct thrombin inhibitor (52). This differs from a previous survey, in which 100% of the centers used UFH (53).

Unfractionated heparin binds antithrombin and increases its activity by 2,000- to 3,000-fold (36). Unfractionated heparin-antithrombin complex inhibits free thrombin and prevents further thrombin generation, whereas thrombin bound to fibrin or subendothelial matrix is unaffected by UFH (12–14, 36). As a result, bounded thrombin will still contribute to thrombin generation, increasing the need for heparin (21).

The advantage of UFH is the low cost, speed in action, familiarity with use, reversibility of its effect through a specific antidote, and anti-inflammatory properties (tissue regeneration, reduction in producing reactive oxygen species, and cardiovascular protection) (12, 13, 36). Furthermore, high heparin infusion dose has been associated with lower hemolysis and mortality rate (12, 50, 54).

Unfractionated heparin binds AT and other plasma proteins, such as acute phase reactant proteins, platelet factor 4, and high molecular weight multimers of vWf (13). The plasma concentration of all these proteins is increased due to the activation of the hemostatic system and the inflammatory response during ECMO, resulting in a reduction of UFH bioavailability and anticoagulant effect (12, 13, 21). In neonates, UFH has a variable effect related to the increase in the volume of distribution, reduction of circulating AT levels, and renal function (12, 13). Moreover, heparin has an age-dependent mechanism of action (4, 55–57). As a result, neonates require greater doses of heparin than adults to achieve thrombin inhibition (21). Therefore, UFH response should be closely monitored through aPTT or ACT (13). In addition, despite being rarely described in neonates, heparin may induce immune-mediated thrombocytopenia due to antibodies against the complex platelet factor 4-heparin (12, 58).

Usually, a heparin bolus of 50–100 U/kg is administered at ECMO start, followed by a continuous infusion at 20–50 U/kg/h (2, 59).

Normal levels of AT are required for UFH to work appropriately, as it inhibits factor IIa, factor Xa, and other serine proteases involved in the coagulation pathway. As stated above, neonates and children have physiologically lower AT levels that reach adult values around 3–6 months of age (8, 13, 16). Moreover, circulating levels of AT seem to decrease over time in ECMO (12, 36, 60–63). For these reasons, according to a survey by Bembea et al. in 2013, 51% of the ECMO centers routinely measure and supplement recombinant human antithrombin (rhAT) (53). In a more recent survey conducted in the US, 73% of the centers reported occasional measuring of antithrombin and 51% empirically administering rhAT (52). Extracorporeal Life Support Organization suggests maintaining AT levels between 80 and 120% (64). However, using rhAT in the neonatal population may increase the bleeding risk, as procoagulant factors are physiologically reduced (13, 36, 60).

Research studies on rhAT replacement therapy showed different and controversial results regarding outcomes (mortality, circuit failure, thrombotic, and hemorrhagic events), diagnostic and therapeutic correlations (UFH infusion, ACT levels, blood product use) (60, 61, 65–69). Indeed, the rhAT administration during ECMO is not routine in the neonatal population, and current clinical practice depends on the single-center protocol.

#### Direct thrombin inhibitors

Direct thrombin inhibitors in ECMO are represented by bivalirudin and argatroban. They have been studied in adult patients, whereas data in newborns and children are scanty and derived from case reports or case series (70–73).

Direct thrombin inhibitors have some advantages compared to UFH, as they can inhibit free and bound thrombin. Indeed, their action is independent of AT, and they do not bind to plasma proteins (12, 36, 74). In addition, a complete thrombin inhibition may reduce coagulation factors consumption, as thrombin-mediated hemostasis activation should be reduced. On the other hand, disadvantages are represented by the lack of inhibition of the contact pathway of the coagulation cascade and their lack of reversibility (12, 36, 74).

Bivalirudin is the most common direct thrombin inhibitor used because of its short half-life (25 min). Still, it has no reversal agent, and its dose must be adjusted in case of renal impairment. Moreover, its dose interval described in the literature is wide (0.05–1.6 mg/kg/h), lacking linearity in the upper side of the therapeutic range when monitored with APTT (4).

However, in recent years, evidence about the use of bivalirudin has been rising in pediatrics. Safety and efficacy have been successfully evaluated in neonatal and pediatric patients as periprocedural anticoagulation or anticoagulation in patients subjected to thrombosis (75–77). Side effects, such as bleeding, were not increased; otherwise, bivalirudin has been associated with more prolonged life of the ECMO circuit and even reduced need for red blood cell transfusions (78, 79). Dosing and monitoring are still a matter of debate. Neonates showed *in vitro* an increased response to bivalirudin in comparison to older children and adults, with a 2-fold reduction in thrombin generation (80).

To our knowledge, a single study exploring the use of bivalirudin in neonatal ECMO has been published. The authors confirmed its safety for thrombosis and bleeding and found that an increased dose of bivalirudin was necessary to maintain stable anticoagulation as a tolerance or tachyphylaxis mechanism developed. Furthermore, there was no significant correlation between monitored bivalirudin dose with APTT and TEG-R (81).

#### Antiplatelet agents and hemostatic adjuncts

Antiplatelet agents, such as acetylsalicylic acid, clopidogrel, and dipyridamole, are frequently used in pediatric VAD patients (82). Therefore, they could be considered therapeutic agents in addition to anticoagulants in ECMO, although thrombocytopenia, common in neonatal ECMO, could increase the risk of bleeding. However, further studies are needed to explore their potential role in neonatal and pediatric ECMO (12, 36).

Antifibrinolytic agents, such as tranexamic acid and ε-aminocaproic acid, are reported to reduce intracranial hemorrhage (ICH) and surgical site bleeding (13, 83, 84). Rational use of these agents may derive from the increased fibrinolysis reported in pediatric and neonatal populations compared to adults (21, 36). However, they have also been associated with more frequent circuit changes and thromboembolic complications, precluding their use in pediatric and neonatal ECMO, except before, during, and after surgeries to reduce the risk of bleeding (13, 36, 85–87).

In some case reports and case series, recombinant activated factor VII (rFVIIa) has been reported to decrease bleeding and packed red blood cells (PRBCs) transfusion need, although with an increased risk of thrombosis (13, 36, 88, 89). Therefore, its use in ECMO remains off-label, and it is not recommended in children, though it could be considered in case of refractory bleeding (13, 36, 90).

Prothrombin complex concentrates (PCC) (3 or 4 Factors PCC) could be considered only in cases of life-threatening and unresponsive bleeding, as it has been studied in a few research studies. In contrast, the administration of activated prothrombin complex concentrate (APCC) has been associated with a chance of fatal thrombosis in an adult patient (13, 91).

#### Transfusional therapy

Blood product transfusions are often administered during neonatal ECMO. The rationale related to the use of blood transfusions products are:

- the need to prime the circuit with PRBCs and fresh frozen plasma (FFP) to avoid hemodilution;
- the need for frequent blood samples to assess coagulation status and to monitor anticoagulation therapy;
- the anemia related to hemolysis;
- bleeding from cannulation and surgical site as a possible complication (92, 93).

Although PRBCs transfusions are common, the established threshold is still a matter of debate, as hemoglobin and hematocrit targets are based primarily on expert opinion (4).

Usually, neonatal and pediatric patients receive 30–105 ml/kg/d of PRBCs (93, 94). Exposure to PRBCs has been associated with increased mortality and morbidity, impacting short- and long-term outcomes (93, 95–99). Indeed, PRBCs may contribute to hemolysis and, in turn, to oxidative stress and thrombotic risk (37, 92, 100). Moreover, oxidative stress is proportional to the volume, and the units of PRBCs transfused (92, 101). In a recent review published in 2018, the authors did not recognize a specific target during ECMO. Still, they suggest that the decision should be based on cardiorespiratory support and oxygen delivery (102).

Platelets and FFP transfusions are performed in high volumes during ECMO, around 25 and 47 ml/kg/d, respectively (92, 103). Their extensive use is driven by the risk of bleeding (104). On the other hand, their use is typically preventive rather than therapeutic, based on blood test alterations and not on evidence of clinical bleeding (105).

Scientific evidence is scarce about indications following laboratory tests for FFP and platelet transfusions (104). The most common thresholds, 80–100 × 10^9^/L for platelets and INR 1.5–1.8 for FFP, are based on expert opinion (4, 92, 104). Depending on the protocol adopted by the single-center, plasma transfusions may also be guided by INR, prothrombin time (PT), aPTT, ACT, antithrombin, TEG R-time, ROTEM A10, and MCF, with a more liberal threshold in case of active bleeding. The laboratory test improvement after FFP or platelet transfusion is mild, both in bleeding and non-bleeding patients (104). In recent years, concern about the safety of platelet transfusion in children and newborns has been rising, as platelet transfusions have been associated with increased mortality (106–109). In conclusion, the transfusional approach is still a matter of debate. Finding a balance between bleeding risk and adverse transfusion effects is challenging, and evidence-based recommendations are lacking.

### How can we monitor hemostasis in neonates on ECMO?

To date, there is not one optimal test to monitor hemostasis and titrate anticoagulation therapy in ECMO (4). Monitoring anticoagulation protocols vary across centers, depending on the expertise of the ECMO team (13). Moreover, monitoring anticoagulation is even more challenging in critically ill newborns, as hemostasis is evolving, and reference ranges are lacking for most tests (12).

A survey from Bembea et al. revealed that 97% of the respondent centers used ACT in monitoring anticoagulation, with most centers using a combination of ACT, APTT, anti-Xa, and thromboelastography (TEG) (13, 53). However, a more recent survey revealed an almost universal application of the anti-Xa assay (90% of the respondent centers) with a decrease in ACT use (65%) (52).

Today a single-test monitoring protocol is not advised, given the complexity of the task. Otherwise, the best combination is still unclear, and multiple tests mean lots of iatrogenic blood loss in a critically ill newborn (Table 2) (12).

**Table 2:** Features of tests available to monitor anticoagulation in ECMO.

**Test**	**Test features**	**Advantages**	**Disadvantages**	**Target**	**Influenced by**
ACT	Point of care Whole blood	Small sample size Low cost Rapid and easy	Poorly related to UFH doses and change Poor correlation with APTT and anti-Xa inhibition	180–220 s	Underlying coagulopathy, platelet dysfunction, AT, age, hemodilution, sample size, temperature
Anti Xa	Lab test Citrated plasma	Direct measure of heparin effect on Xa	High cost Not ready available Expertise needed No role in determining the hemostatic potential	0.3–0.7 IU/ml	Hyperbilirubinemia, plasma-free Hb levels, hypertriglyceridemia, AT levels, assay type
APTT	Lab test Citrated plasma	Low cost Widely spread Readily available	Often prolonged in newborn Poor correlation between APTT and anti-Xa Often overestimates heparin activity	Ratio 1.5–2.5 times baseline	Hyperbilirubinemia, hyperlipidemia, anti-phospholipid antibodies, increased C reactive protein, liver disease, UFH contamination, hemodilution
TEG/ROTEM	Point of care Whole blood	Small sample size Rapid and easy Reflect whole hemostasis Guide transfusion need	Lack of neonatal reference ranges	R time in kaolin 2–3-fold longer than R time in heparinase (R time in kaolin 15–25 min)	Reagent and plasma-free Hb

AT, antithrombin; Hb, hemoglobin; s, seconds; UFH, unfractionated heparin.

A disadvantage common to all coagulation assays is the difference in the reagents and coagulation analyzers used in the laboratories, which makes them not well-comparable and standardized (6). As a result, correlations between laboratory test results and complications, such as thrombosis, bleeding, or mortality, have not been found in various studies (54, 110–113).

#### Activated clotting time

The oldest and best-known test to monitor anticoagulation is ACT, which measures the intrinsic and common pathway of the coagulation cascade (21).

Activated clotting time is a rough but straightforward bedside test performed on whole blood to assess anticoagulation adequacy (14). It measures the time for whole blood to clot when activated by kaolin, celite, or glass beads (12). It is low cost, requires a small sample size (2–3 whole blood drops), is easy to perform, and may be used even during transport (2).

Activated clotting time range targeted is usually between 180 and 220 s, with the expected rate of heparin infusion 20–50 U/kg/h (14, 21). Depending on the ACT result obtained, UFH infusion may be adjusted, and the ACT re-checked in an hour (13).

Activated clotting time remains one of the most common tests used to monitor anticoagulation in ECMO, despite its results being influenced by patient underlying coagulopathy, platelet dysfunction, AT, age, hemodilution, sample size, and temperature (21). Due to all these influencing factors, reproducibility is scarce, especially in neonates where hemostasis is continuously developing (14).

Another disadvantage of ACT is its accuracy, which decreases with the ongoing time of ECMO. However, its reliability improves when adequate clotting factor levels are maintained during the procedure (21).

To note, if the ACT is out of range with an adequate heparin infusion rate, other factors influencing hemostasis must be searched with other tests. Activated clotting time values correlate poorly with heparin levels, anti-Xa, or APTT (4, 14, 21). Activated clotting time is the test with the lowest correlation with heparin dose and is the least affected by changes in heparin doses compared to anti-Xa and APTT, especially in low heparin doses as in ECMO (113–116). In addition, a weak correlation has been found between ACT and platelet count, with the highest ACT values when the platelet count is below 100.000/mmc (111, 113).

Other ACT tests such as the i-STAT ACT are now available. In addition, ACT derived from POC instruments such as the TEG with the TF activation may be a good test for the future, despite its use not being universally spread yet (21).

In summary, the ACT is an excellent crude method to obtain immediate information about the anticoagulation of the circuit. Still, it is insufficient to monitor anticoagulation in ECMO correctly, so other tests should be considered for addiction (Table 2) (21).

#### Anti-factor Xa

The anti-factor Xa test is now the most popular test for UFH and low molecular weight heparin (LMWH) monitoring in many ECMO centers (14, 52). It measures the inhibition of factor Xa by heparin in plasma, detecting the rate of factor Xa inactivation by the heparin/antithrombin complexes (12, 110, 113, 117).

Unlike the ACT, which is influenced by all the other factors with a potential impact on coagulation, anti-Xa levels measure the heparin effect or concentration and the inhibition of FX conversion. This explains the weak correlation between anti-Xa levels and ACT in adult and pediatric/neonatal ECMO (4, 111). However, it has been found to have the strongest correlation with heparin dose compared to ACT, APTT, TEG, and AT levels (111, 113, 114). Otherwise, it is not affected by other parameters with hemostatic impacts, such as FVIII or fibrinogen levels (113).

The anti-factor Xa test can be performed in the absence or presence of AT. The first case measures the heparin effect, while the second measures the heparin concentration. The effective range is between 0.3 and 1.1 IU/ml, depending on the single-center, with the most common goal range used 0.3–0.7 IU/ml (2, 14, 52). In neonates, as they have low levels of AT, an anti-Xa assay in the absence of AT is more representative of what happens *in vivo*, otherwise may overestimate heparin activity and mask AT deficiency (12, 52).

To note, anti-Xa has poor reliability in patients with hyperbilirubinemia, increased plasma-free hemoglobin levels, and hypertriglyceridemia and is influenced by AT levels and assay type (2, 4). Other disadvantages are the training required to be performed, its high costs, and the time to run the test (114). Moreover, it has no role in evaluating thrombin generation, so it should be integrated with other tests (Table 2) (12).

Anti-Xa-based monitoring of UFH protocols showed that higher doses of heparin are needed in infants compared to older children to obtain therapeutic anti-Xa levels (52, 110). In addition, a more stable heparin dosing, with few “out of range” results, without increased complications, has been found with anti-Xa-based protocol compared to ACT-based protocol (118).

#### Activated partial thromboplastin time and prothrombin time

Activated partial thromboplastin time is widely recognized as a test to monitor heparin therapy in adults, even if it is poorly reliable in the acute management of ECMO (14, 21, 117).

Activated partial thromboplastin time represents the clotting time of recalcified, platelet-poor citrated plasma when activated by an intrinsic pathway activator (12, 14). Different methods and analyzers are used to determine APTT, influencing the sensitivity to heparin, and the reference ranges to use, making the test less comparable across centers (13).

Prothrombin time and activated partial thromboplastin time are often prolonged in newborns, regardless of ECMO. Therefore, they are unreliable for studying *in vivo* hemostasis in neonatal age (16, 18). In addition, they have low prediction capability in clinically significant bleeding (19, 22, 23). The APTT response to heparin also varies with age, with the more substantial prolongation of APTT in younger children exposed to the same heparin amounts as the older ones (4, 56, 57). Poor correlation between APTT and anti-Xa has been reported, too (119, 120). In other studies, the correlation between APTT and anti-Xa was moderate, but APTT often overestimated heparin activity (110).

Moreover, APTT is influenced by hyperbilirubinemia, hyperlipidemia, anti-phospholipid antibodies, and increased C reactive protein (12).

In adults, APTT ratio values 1.5–2.5 times above baseline moderately correlate with a heparin concentration, preventing thrombus formation (14, 21).

In summary, APTT may be used in monitoring anticoagulation in adults because it correlates well with anti-factor Xa levels, less so in children and neonates (Table 2) (14).

Prothrombin time is a citrate plasma clotting time that investigates the clotting factors of the extrinsic and common pathway of the coagulation cascade through factor VII activation by TF and phospholipid. Prothrombin time has a strong correlation with coagulation factors levels but minimal correlation with heparin, AT, and factor XII (113). It is the most specific test for changes in clotting factors of the extrinsic and common pathways (113). It may be helpful in ECMO as it may detect the need for clotting factors supplementation in case of bleeding with normal heparin activity (Table 2).

#### Thromboelastography and rotational thromboelastometry

The use of viscoelastic coagulation tests (VCTs), such as thromboelastography (TEG) and rotational thromboelastometry (ROTEM), is widespread for anticoagulation monitoring in ECMO (Table 2). In a recent survey conducted in the US, 41% of the centers reported using a viscoelastic test to monitor anticoagulation (52).

They have the advantage of dynamically examining the clotting process, from detecting the first fibrin filaments to the clot lysis (21). Moreover, VCTs provide information about the coagulation factors, platelet functions and number, platelet-fibrin interaction, fibrinogen levels and activity, and fibrinolysis (14). Like ACT, they are bedside techniques, making the result available in real-time (12).

**Figure 1:**
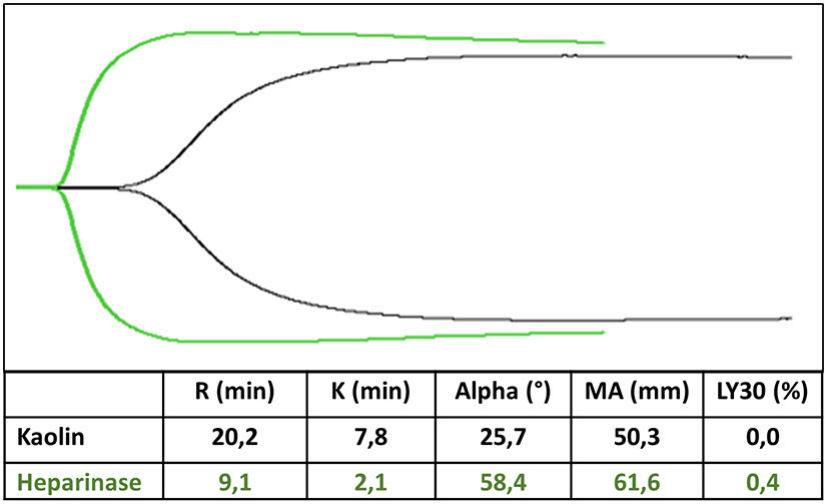
TEG trace in kaolin (black trace) and heparinase (green trace). R-time is prolonged 2–3-fold in kaolin compared to heparinase, whereas MA is unaffected by heparin.

Viscoelastic coagulation tests are represented as a flat line corresponding to the liquid phase of the whole blood. Then, the line diverges into two lines with increasing amplitude until the maximum clot firmness is reached. Finally, the two lines converge simultaneously with the clot lysis (Figure 1) (121). All these steps are described by the different TEG/ROTEM parameters. They may indicate which aspect of the hemostatic process is disrupted, with a possible therapeutic approach to correct the coagulopathy.

*Thromboelastography reaction time (R)/ROTEM Clot formation Time (CT)* reflects the time from the test beginning to the first detection of fibrin filaments. The *alpha angle and coagulation (K) time* describe how fast the clot grows and depends on clotting factors, platelets, and fibrinogen levels. *TEG Maximal Amplitude (MA)/ROTEM Maximal Clot Firmness (MCF)* represents the platelets and fibrinogen activity. *TEG Lysis (LY) 30/ROTEM Clot Lysis Index (CLI) 30* describes the fibrinolysis 30 min after *MA/MCF* (121, 122).

Rotational thromboelastometry and thromboelastography provide the same information, even with different parameter names, although the results are not interchangeable as they use different methodology and laboratory assays (123).

Thromboelastography can be performed with whole fresh blood that must be analyzed within 4–6 min, whereas if citrated blood is used, the test can be delayed for a few hours, albeit with slightly different results (121).

**Figure 2:**
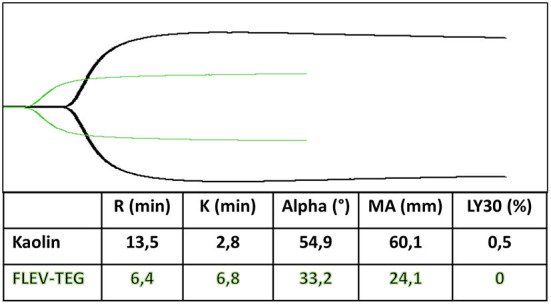
Functional fibrinogen test (FLEV-TEG) (green trace) shows the platelet contribution to MA parameter compared to kaolin test (black trace).

Activators are frequently utilized in VCTs to faster the process. Kaolin is the most used with TEG; its ROTEM equivalent is the INTEM, which uses ellagic acid and phospholipids to explore the contact-dependent coagulation pathway. Rotational thromboelastometry (ROTEM) EXTEM uses TF to analyze the extrinsic coagulation pathway. At the same time, rapid TEG activates coagulation through a combination of TF and kaolin to allow faster assessment of the hemostatic status (121, 124). Adding heparinase (Heparinase in TEG, HEPTEM in ROTEM) allows a heparin-free curve in patients subjected to anticoagulation with UFH (21, 121). Moreover, the functional fibrinogen test (FLEV-TEG in TEG, FIBTEM in ROTEM) allows for measuring the amount of fibrinogen that contributes to the clot strength. They block the platelet contribution to the MA or MCF parameter by TF and abciximab, a GPIIb/IIIa inhibitor (FLEV-TEG), and TF and cytochalasin D, an actin polymerization inhibitor (FIBTEM) (Figure 2). Their use permits discrimination between the deficit of fibrinogen and the deficit of platelets and decides which one must be administered to the patient (125–130).

Viscoelastic coagulation tests are successfully used in adult critical care settings to guide transfusion practice, improving outcomes (121, 131, 132). In NICU, TEG and ROTEM are attractive owing to the peculiarity of the hemostatic system of the newborn, especially preterm, to assess the coagulation status and potential to drive FFP or platelets administration (19, 22, 92, 124, 133–144).

Reaction and clot formation (R and CT) time are the most important parameter when using TEG or ROTEM in ECMO, as it refers to the time needed to obtain the first fibrin filaments (Figure 1). They are influenced mainly by clotting factors and by treatment with heparin of vitamin K antagonists (121).

Therefore, anticoagulation management is usually based on R or CT time, comparing the values obtained with or without heparinase (123).

In our experience, the best way to monitor anticoagulation with TEG is to perform a heparinase TEG and a kaolin TEG simultaneously, with *R time* in kaolin 2–3 fold longer than *R time* in heparinase (*R time* in kaolin 15–25 min) (2, 21). The ratio between *R time* in kaolin and *R time* in heparinase drives the need to increase or decrease heparin infusion (2). Maximal amplitude parameter, which describes the maximum amplitude of the TEG trace, is a measure of platelets and fibrinogen concentration and function and may have a role in determining the need for fibrinogen or platelets administration when the functional fibrinogen test is performed (2). The same tests may be performed with ROTEM.

Studies have demonstrated a weak correlation between TEG and APTT/ACT and a strong correlation between *MA* and platelet count (145). In addition, qualitative platelet dysfunction during ECMO has been demonstrated in a retrospective pediatric study using Platelet-Mapping TEG. Still, the correlation between this alteration and bleeding risk is yet to be determined (146).

In a retrospective study, a reduced MCF of INTEM, EXTEM, and INTEM with heparinase showed a correlation with increased thrombotic risk. Coagulation factors and platelet consumption may explain this finding during ECMO. No correlation was found between ROTEM parameters and bleeding complications (128).

Clot formation time on INTEM and HEPTEM was found to have a moderate-strong correlation with aPTT and HaPTT for children receiving bivalirudin for anticoagulation (123).

In adults, the use of POC tests to monitor anticoagulation has been demonstrated to provide reliable and timely information about the risk of bleeding, with a good correlation with traditional coagulation tests (147). They reflect the patient's hemostatic status in mechanical circulatory support, monitor the anticoagulation and anti-aggregation therapy, and their use reduces the risk of thromboembolic and bleeding complications (15). A retrospective study on neonates affected by congenital diaphragmatic hernia revealed improved goal-directed blood product transfusions and reduced bleeding complications, especially hemothorax requiring chest tube placement or thoracotomy (148).

Further studies to standardize TEG and ROTEM are required, as reference ranges for neonatal and pediatric populations are not well-established, as well as optimal target values for ECMO patients (52, 59, 122).

## Future perspectives and conclusion

Due to the complexity of the hemostatic system of the newborn and the impact of extracorporeal circulation on the hemostatic balance, anticoagulation management is a real challenge during neonatal ECMO.

Unfractionated heparin remains the mainstay of treatment, but other agents, such as antiplatelets and direct thrombin inhibitors, are of great interest and could be considered in the future (12, 36). In addition, newer anticoagulant agents, such as factor XIa and XIIa inhibitors, are now in the early stage of research studies. They might be promising in the future, as they can uncouple the antithrombotic effect from the anti-hemostatic effect (149–151).

The best strategy to monitor and titrate anticoagulation is still unknown, but in recent years, different and more reliable tests have substituted the traditional ACT and APTT. A combination of multiple techniques is probably the best option, as they explore various aspects of the coagulation status. In our experience at the neonatal ECMO center, TEG may provide helpful real-time information to drive the heparin infusion and the need for blood product transfusions, besides other tests. Of course, further studies are needed to clarify the role of every single test in the overall management and the best combination to achieve a safe balance between hemostasis and thrombosis during ECMO to obtain a shared protocol across the centers.

## Author contributions

VC, GR, GSA, IA, SGu, FMa, GCe, AT, MC, AA, SGh, and GCa contributed to the study's conception and design. VC, GR, GSA, IA, SGu, FMa, SGh, and GCa contributed to the study's methodology, investigation, and data curation. VC, GR, GSA, IA, SGu, FMa, SGh, and GC wrote the original draft preparation of the manuscript. VC and GR contributed equally to this work and share the first authorship, having the right to list their name as first in their Curriculum Vitae. GCe, AT, MC, AA, SGh, GCa, and FMo provided extensive critical revision. All authors contributed to the manuscript's critical revision, read and approved the submitted version.

## Funding

This study was (partially) funded by the Italian Ministry of Health—Current research IRCCS.

## Conflict of interest

The authors declare that the research was conducted in the absence of any commercial or financial relationships that could be construed as a potential conflict of interest.

## Publisher's note

All claims expressed in this article are solely those of the authors and do not necessarily represent those of their affiliated organizations, or those of the publisher, the editors and the reviewers. Any product that may be evaluated in this article, or claim that may be made by its manufacturer, is not guaranteed or endorsed by the publisher.

## Abbreviations

ACT: activated clotting time
APTT: activated partial thromboplastin time
AT: antithrombin
CFT: clot formation time
CHD: congenital heart disease
CLI: clot lysis index
ECMO: extracorporeal membrane oxygenation
ECPR: extracorporeal cardiopulmonary resuscitation
ELSO: Extracorporeal Life Support Organization
FFP: fresh frozen plasma
ICH, intracranial hemorrhage K time: coagulation time
LMWH: low molecular weight heparin
LY: clot lysis
MA: maximal amplitude
MCF: maximal clot firmness
MCV: mean corpuscular volume
NICU: neonatal intensive care unit
PMEA: poly-2-methoxy-ethyl acrylate
PRBCs: packed red blood cells
PT: prothrombin time
RBCs: red blood cells
ROTEM: rotational thromboelastometry
R time: reaction time
TEG: thromboelastography
TF: tissue factor
UFH: un-fractioned heparin
US: United States
VAD: ventricular assist device
VCT: viscoelastic coagulation test
vWf: von Willebrand factor.
